# Study on the impact of common air pollution indicators on tuberculosis incidence in high-TB-burden countries worldwide

**DOI:** 10.3389/fpubh.2025.1628290

**Published:** 2025-11-03

**Authors:** Minli Chang, Zhifei Chen, Xiaodie Chen, Xilong Du, Nana Zhang, Dongmei Lu, Liping Zhang, Yanling Zheng

**Affiliations:** ^1^College of Public Health, Xinjiang Medical University, Urumqi, China; ^2^Institute of Medical Engineering Interdisciplinary Research, Xinjiang Medical University, Urumqi, China; ^3^College of Medical Engineering and Technology, Xinjiang Medical University, Urumqi, China; ^4^Center of Pulmonary and Critical Care Medicine, People’s Hospital of Xinjiang Uygur Autonomous Region, Urumqi, China

**Keywords:** tuberculosis (TB), pulmonary tuberculosis (PTB), air pollution, generalized additive models (GAMs), interactive effects of air pollutants

## Abstract

**Objective:**

To explore the effect of air pollution on tuberculosis (TB) in multiple countries and to provide a scientific reference for air pollution treatment and tuberculosis prevention and control.

**Methods:**

Spearman’s correlation analysis and generalized additive models of air pollution indicators and the annual incidence of tuberculosis in the top 20 countries with global tuberculosis incidence in 2021 were conducted to investigate the association effect between air pollution and tuberculosis incidence globally from 1990 to 2020.

**Results:**

The severity of the global TB epidemic in 2021 varied widely among countries, and Spearman’s correlation analyses showed a positive correlation between TB incidence and Household air pollution (HAP; r_HAP_ = 0.476) and negative correlations between NO_2_, O_3_, PM, and Gross domestic product (GDP) and TB incidence (r_NO2_ = −0.622, r_O3_ = −0.419, r_PM_ = −0.323, and r_GDP_ = −0.477). By examining the effects of influencing factor interactions on the development of tuberculosis, it was found that at HAP < 0.2, TB incidence tended to increase with increasing NO_2_, and the risk of TB incidence increased at lower NO_2_ (2–6 ppb) and O_3_ < 40 $\mathrm{\mu g}/m^{3}$.

**Conclusion:**

There is a synergistic amplification of the increase in TB incidence by HAP, O_3_, GDP, and NO_2_, with low NO_2_ concentrations, low HAP, and high O_3_ conditions favouring TB. The incidence of TB is adversely affected by air pollution to varying degrees across countries; therefore, countries can target preventive measures to reduce the risk of TB.

## 1 Introduction

Tuberculosis (TB) is an ancient chronic infectious disease caused by *Mycobacterium tuberculosis* (MTB) that spreads through airborne droplets from coughing, sputum, and loud talking. Inhalation of these droplets can lead to Latent TB infection (LTBI) (1). *Mycobacterium tuberculosis* predominantly infects human lungs, and it is estimated that approximately a quarter of the global population is infected with *Mycobacterium tuberculosis*. Although the vast majority of latently infected individuals do not progress to active tuberculosis, about 5 to 15% of *Mycobacterium tuberculosis* latently infected individuals develop active tuberculosis during their lifetime (2), with 90% of them being adults, and more of them being males than females (3). According to the Global Tuberculosis Report 2024, tuberculosis (TB) replaces the new crown (COVID-19) to regain its position as the leading cause of death due to a single infectious agent in the world in 2023, with more than 10 million people falling ill each year (4), and the number of people falling ill globally growing from 10.1 million to 10.8 million between 2020 and 2023. The highly contagious nature of the disease due to its uncontrollable route of transmission and the recurrence rate after treatment in cases of inadvertent infection are the main reasons why TB has been with us for thousands of years (5). Air pollution is the world’s top environmental risk factor for human health (3), and in recent years, a large number of studies at home and abroad have shown that there is a certain correlation between the incidence of tuberculosis and air pollutants. According to the Global Status of Air Report 2024, air pollution is responsible for about 8.1 million deaths globally in 2021, accounting for about 1/8 of all global deaths, and it is the world’s 2nd largest risk factor leading to death (6).

Air pollution is a global issue that significantly impacts both the atmosphere and human health. Industrialization and urbanization have increased pollutant emissions. Their widespread diffusion and invisibility during spread pose direct and indirect threats to respiratory health (7). Air pollutants such as Household air pollution (HAP) from solid fuels, Nitrogen dioxide (NO_2_) pollution, Ambient ozone (O_3_) pollution, and Ambient particulate matter (PM) pollution increase the risk of TB development through multiple pathways of immunosuppression, tissue damage, and pathogen synergy. PM is a key TB risk factor due to its small size, light weight, toxicity, and ability to carry harmful substances. Metals (e.g., Fe and V) and organic components (e.g., PAHs) in PM impair macrophage phagocytosis and lysosomal enzymes, boosting the survival of intracellular TB bacteria (8). Simultaneously, PM exacerbates inflammation but suppresses protective immunity by activating the NLRP3 Inflammasome and releasing IL-1β and IL-18 (9). PM also interferes with the antigen-presenting function of Dendritic Cells (DCs), reduces CD4^+^ T-cell differentiation and Interferon-*γ* (IFN-γ) secretion, and impairs the control of reactivation of latently infected tuberculosis bacilli (8). The atmospheric pollutant NO_2_ acts as a precursor to near-surface O_3_ pollution and contributes to TB progression through multiple pathways. In the respiratory tract, NO₂ generates Reactive Nitrogen Species (RNS), such as peroxynitrite, in the humid environment of the respiratory tract, which disrupts epithelial cell structure, inhibits mucus-ciliary scavenging, decreases macrophage inducible Nitric Oxide Synthase (iNOS) activity, and reduces antimicrobial Nitric oxide (NO) production, leading to a decrease in *M. tuberculosis* clearance capacity (10). In addition, NO₂ exposure stimulates airway epithelial cells to continuously release pro-inflammatory factors, such as TNF-*α* and IL-6, leading to chronic inflammation and destruction of the microenvironment of lung tissues. At the same time, it inhibits the secretion of IFN-*γ* and promotes the T helper 2 cell response (Th2), e.g., IL-4 and IL-13, exacerbating airway hyperresponsiveness and tissue damage (11). O_3_ exposure deteriorates lung function, exacerbates airway inflammatory responses, affects lung ventilation (12), and increases the risk of tuberculosis. O₃ reacts with respiratory epithelial cell lipids, generating large quantities of reactive oxygen species (ROS), e.g., Hydroxyl Radical, depleting Glutathione (GSH) and Superoxide Dismutase (SOD), triggering DNA damage and apoptosis (13). In addition, O₃ downregulates the chemokine CXCL10 and reduces the migration of T cells and NK cells to the site of infection, while recruiting over-activated neutrophils via CCL7 and CXCL10, releasing elastase and myeloperoxidase, and exacerbating lung tissue damage rather than being effective bactericidal (13). The core source of indoor air pollution is the incomplete combustion of solid fuels, which produces black carbon particles and PAHs, e.g., benzo(a)pyrene (BAP), which has a stronger oxidative stress-inducing capacity and endothelial dysfunction effects than PM (14). On the one hand, CO produced by combustion competitively binds to haemoglobin, resulting in tissue hypoxia, leading to impaired energy metabolism in macrophages and thus reduced bactericidal capacity (15). On the other hand, PAHs, through activation of the Aromatic Hydrocarbon Receptor (AhR), inhibit T cell differentiation, reduce interferon-*γ* (IFN-γ) secretion, and induce a Th17-type inflammatory response, exacerbating lung inflammation but reducing protective immunity (16).

Due to inadequate infrastructure and high energy prices, three billion people worldwide rely on polluting fuels and technologies for home cooking and heating. However, studies have pointed out that indoor air pollution is one of the major risk factors for various adverse health outcomes (including premature death) worldwide (17). Ipek and Ipek analysed the micro-longitudinal dataset of income living conditions in Turkey from 2014 to 2017 using a random-effects panel discrete ordered model and found that factors such as age, gender, and indoor air pollution have a negative impact on health status, and that even high-income households are adversely affected by indoor air pollution because they do not have access to clean energy (18). Lee et al. (19) estimated the global, regional, and national health burdens associated with exposure to indoor air pollution and found that indoor air pollution was positively associated with tuberculosis (RR 1.26, 95% CI 1.08–1.48). Blount et al. (20) analysed the association between latent tuberculosis and common sources of urban indoor air pollution in Southeast Asia and found a strong association between exposure to indoor air pollutants and latent tuberculosis infection. Feng et al. (21) analysed the relationship between long-term exposure to air pollutants (2005–2017) and the risk of tuberculosis (TB) in the Beijing-Tianjin-Hebei region using a combined Poisson generalized linear regression-distributed lag nonlinear model, and the results of the study revealed potential associations between outdoor exposures to particulate matter with aerodynamic diameters of ≤2.5μm (PM_2.5_), NO_2_, and O_3_ and the risk of PTB. Cao et al. (22) used a generalized additive model with Poisson distribution to analyse the effects of air pollutants and meteorological factors on the incidence of new tuberculosis patients in Yulin City during 2017–2021 and found that there was a weak correlation between elevated air pollutant concentrations and the incidence of tuberculosis. Dongmei et al. (23) used a distributional lag nonlinear model to explore the effect of exposure to gaseous pollutants on the risk of tuberculosis in Nantong City from 2014 to 2018 and found that outdoor NO_2_ exposure increased the risk of developing tuberculosis. A study by Alkhanani used Python to analyse four years of Air Quality Index (AQI), O_3_, NO_2_, respiratory disease incidence, and socio-economic factor data from 27 countries. The results of the study emphasized the close association between air pollutants and respiratory diseases, as well as the significant influence of socio-economic factors on these relationships (24). A study in Chengdu City found a positive correlation between outdoor PM_10_, SO_2_, and NO_2_ exposure and the number of TB cases (25). Liu et al. (26) used penalized multivariate Poisson regression models to assess the impact of short- and long-term ambient SO_2_, NO_2_, carbon monoxide (CO), O_3_, and PM_2.5_ exposures on the development of active tuberculosis and mortality in seven cities from 2013 to 2017, and the results of the study showed that SO_2_ and PM_2.5_ exhibited more consistent and stronger associations with tuberculosis-related outcomes. Deng et al. (27) analysed PM_2.5_, in Shenyang City, China, during 2014–2018. And O_3_ as potential joint effects and found that PM_2.5_ and O_3_ had a synergistic effect with a synergistic index SI of 2.372, emphasizing the importance of the synergistic effect between O_3_ and PM_2.5_ in exacerbating respiratory mortality. Liang et al. (28) explored the relationship between atmospheric pollutants and hospitalization for respiratory diseases and found that, in addition to CO, PM_10_, PM_2.5_, SO_2_, NO_2_, and O_3_ increased the risk of hospitalization for respiratory diseases. Song et al. (29), in an investigation of the effect of NO_2_ exposure on the development of TB disease, found that NO_2_ was associated with a reduced risk of developing drug-resistant tuberculosis. From the above studies, it can be concluded that relevant studies have confirmed that air pollutants may affect the survival and transmission process of *Mycobacterium tuberculosis*, but the assessment of air pollution and the risk of tuberculosis incidence is more complicated due to the need to take into account other influences, such as social factors, such as the level of economic development (30). Since a number of air pollutant concentrations do not satisfy a linear relationship with TB incidence, this study was analysed using a generalized summation model, which is a semiparametric regression model that can flexibly fit a wide range of complex data patterns in order to reveal the complex relationship between incidence and these factors (31).

A large body of research evidence suggests that indoor air pollution, NO_2_, O_3_, and PM have emerged as important risk factors for TB infection, active TB disease, and TB mortality (32). The burden of disease due to environmental risk factors is an avoidable disease burden (33), and many TB-endemic countries tend to have high levels of air pollution, but research related to air pollution and TB is still in its infancy (34). Knowledge of exposure risk levels and trends in air pollution is important for determining public health policies to reduce the burden of TB caused by it. Therefore, the aim of this study was to systematically analyse the trend of changes in TB incidence due to exposure to indoor air pollution, NO_2_, O_3_, and PM during 1990–2000 in the top 20 countries with global TB incidence in 2021 using a generalized additive model. It is hoped that this study will complement and update previous studies and inform the development of effective TB prevention policies to reduce public health risks and promote health and well-being for all.

## 2 Materials and methods

### 2.1 Data source

The air pollutants and socio-economic indicators covered in this study are globally standardized measurements covering the following air pollution types: PM, NO_2_, O_3_, and HAP. Exposure to PM ($\mathrm{\mu g}/m^{3}$) is defined as the population-weighted annual average mass concentration of PM per cubic metre of air in the Global Burden of Disease Study 2021 (GBD 2021), which includes both natural sources, such as sand and dust, and anthropogenic sources, such as industrial emissions, and is derived from the data set “Ambient particulate matter pollution” from GBD 2021, using a chemical transport model to simulate air quality. From the GBD 2021 “Ambient particulate matter pollution” dataset, simulated using a chemical transport model (35); NO_2_, an air pollution marker strongly influenced by motor vehicle emissions, was added as a new risk factor in GBD 2021, representing the annual average ground-based monitoring concentration, measured by chemiluminescence in ppb (parts per billion). The data are from the GBD 2021 “Nitrogen dioxide pollution” dataset, which combines satellite remote sensing, such as the TROPOspheric Monitoring Instrument (TROPOMI), with ground-based monitoring data. It reflects the level of emissions from transportation and industry and adds another dimension to the air pollution situation (36). O₃ exposure is taken as the seasonal average of the daily 8-h maximum concentration in ppb (35), which reflects the intensity of photochemical pollution and is derived from the GBD 2021 “Ambient ozone pollution” dataset based on a global monitoring network such as the NASA Aura satellite; Estimates of exposure to HAP are based on estimates of the proportion of individuals and the proportion of households in each country using solid fuels, including coal, wood, charcoal, dung, and agricultural residues, in combination with PM exposure from the GBD 2021 “Household air pollution from solid fuels” dataset, with estimates of the percentage of households using solid cooking fuels provided for each country’s representative data point, reflecting the level of PM exposure in households using solid fuels, with values ranging between 0 and 1. Data from the GBD 2021 “Household air pollution from solid fuels” dataset, where each nationally representative data point provides an estimate of the percentage of households using solid cooking fuels (35), reflecting the level of exposure to PM in households using solid fuels, with values ranging from 0 to 1 and being dimensionless, with higher values indicating more severe exposure; The GDP data for this study come from the World Bank National Accounts data and the Organization for Economic Cooperation and Development National Accounts data files. The main data used are GDP per capita normalized by population (GDP divided by mid-year population), which gives an indication of the level of economic development. Data are expressed in current United States dollars (USD).

All air pollutant data in this study were obtained from the Global Burden of Disease Study 2021 database (GBD 2021); all data were corrected by the GBD team’s Bayesian modelling, which corrected for spatial heterogeneity, such as urban–rural differences, and temporal trends, such as bias due to technological advances in monitoring, and the uncertainty in the air pollutant exposure estimates was quantified by the 95% Uncertainty Interval (UI) and averaged for inclusion in the model; GDP data were used from World Bank official statistics, and the study investigated the relationship between the incidence of TB and air pollution indicators and GDP per capita in the top 20 countries in the world in 2021, as given by GBD 2021, covering the following countries: Angola, Bangladesh, China, DR Congo, Ethiopia, India, Indonesia, Kenya, Mozambique, Myanmar, Nigeria, Pakistan, the Philippines, the Russian Federation, South Africa, Thailand, Uganda, Tanzania, Vietnam, and Zimbabwe. Data are standardized at the national level, and inter-country comparability can be ensured.

### 2.2 Statistical method

In this study, Excel 2021 software was applied for data preprocessing, R 4.1.3 software was used for descriptive statistical analysis of the data, the mgcv software package was used to fit the generalized additive model to analyse the effect relationship between the influencing factors and the incidence of tuberculosis, and Spearman’s correlation analysis of the influencing factors and the incidence of tuberculosis was carried out by using SPSS 25 software. The test level was 0.05.

The core idea of Generalized additive models (GAMs) is to decompose a model into parts, each of which describes the relationship between a set of variables. Each part is ‘additive’, meaning that each part contains only one variable or a function of a variable, and their sum is the complete model. This decomposition allows GAMs to handle high-dimensional data and avoids the problem of overfitting. Since the incidence of TB in this study is a small probability event relative to the total population and its actual distribution is close to the Poisson distribution, the Poisson distribution is used as the distribution cluster of the regression model, which corresponds to the generalized additive model that takes the log link function to analyse the effects of air pollution indicators and GDP on the incidence of TB in 20 high-incidence countries. The relationship between air pollution indicators and GDP and TB incidence is non-linear; therefore, the influencing factors are included in the GAM model with a natural spline function. The basic model is as follows:


(1) $$\log[E(y_{i})]=\text{Country}+s(x_{j})+\varepsilon$$

(2) $$\begin{array}{l} \log[E(y_{i})]=\text{Country}+s(\mathrm{HAP})+s({\mathrm{NO}}_{2})\\ +s(O_{3})+s(\mathrm{PM})+s(\mathrm{GDP})+\varepsilon \end{array}$$

(3) $$\log[E(y_{i})]=\text{Country}+s(\mathrm{HAP},{\mathrm{NO}}_{2})+\ldots +s(\mathrm{PM},\mathrm{GDP})+\varepsilon$$

where $y_{i}$ is the incidence rate of tuberculosis in year i; $E(y_{i})$ is the expected value of the incidence rate of tuberculosis in year i; s() is the smoothing spline function, which is used to fit the variable that has a complex, non-linear relationship with the dependent variable; Countryis the control factor; and $x_{j}$ represents the influencing factor of $\mathrm{HAP},NO_{2},O_{3},\mathrm{PM},\mathrm{GDP}$; and ε is the residual of the model. The effect of model fit was tested using GAM check() to prevent overfitting. Confounding factors such as population density and healthcare resources were not adjusted for in this study because the GBD data already included sociodemographic covariates.

First, the independent effects of different influencing factors on TB incidence were analysed by fitting GAMs according to Equation 1. On this basis, the indicators that have significant effects on the changes of TB incidence were screened out, and the interaction effects of all the influencing factors interacting on the incidence of TB were further analysed by the proposed GAMs according to Equation 2. According to Equation 3, GAMs were proposed to explore the effect of two-by-two interaction on the incidence of tuberculosis, and the results of GAMs were analysed by Effective Degrees of Freedom (EDF), Reference Degrees of Freedom (RDF), F-statistic value, *p*-value, Adjusted R-squared (R^2^), Deviance Explained (DVE), Generalized Cross-Validation Value (GCV), and Akaike Information Criterion (AIC) parameters for characterization. Among them, the degree of freedom is used to judge the linear or non-linear relationship between the influencing factors and the response variables: a degree of freedom of 1 indicates that the influencing factors and the response variables are linear; a degree of freedom greater than 1 indicates that the influencing factors and the corresponding variables are non-linear, and the larger the value, the stronger the non-linear relationship. In this paper, the significance level of the statistical results of the model is taken as 0.05 (*p* value of <0.05). The F-statistic value is used to judge the relative importance of the factors; the larger the value, the more factors there are in relative terms. R^2^, DVE, GCV, and AIC are used to judge the fitting effect of the model. DVE indicates that the variance of the response variable explained by the model accounts for a proportion of the total variance, and GCV is a trade-off indicator between complexity and goodness of fit. GCV is a trade-off indicator between model complexity and goodness of fit. When R^2^ and DVE are larger, and GCV and AIC are smaller, it represents a better fitting effect, and the model has better generalization ability.

## 3 Results

### 3.1 Epidemiological characteristics of tuberculosis

**Figure 1:**
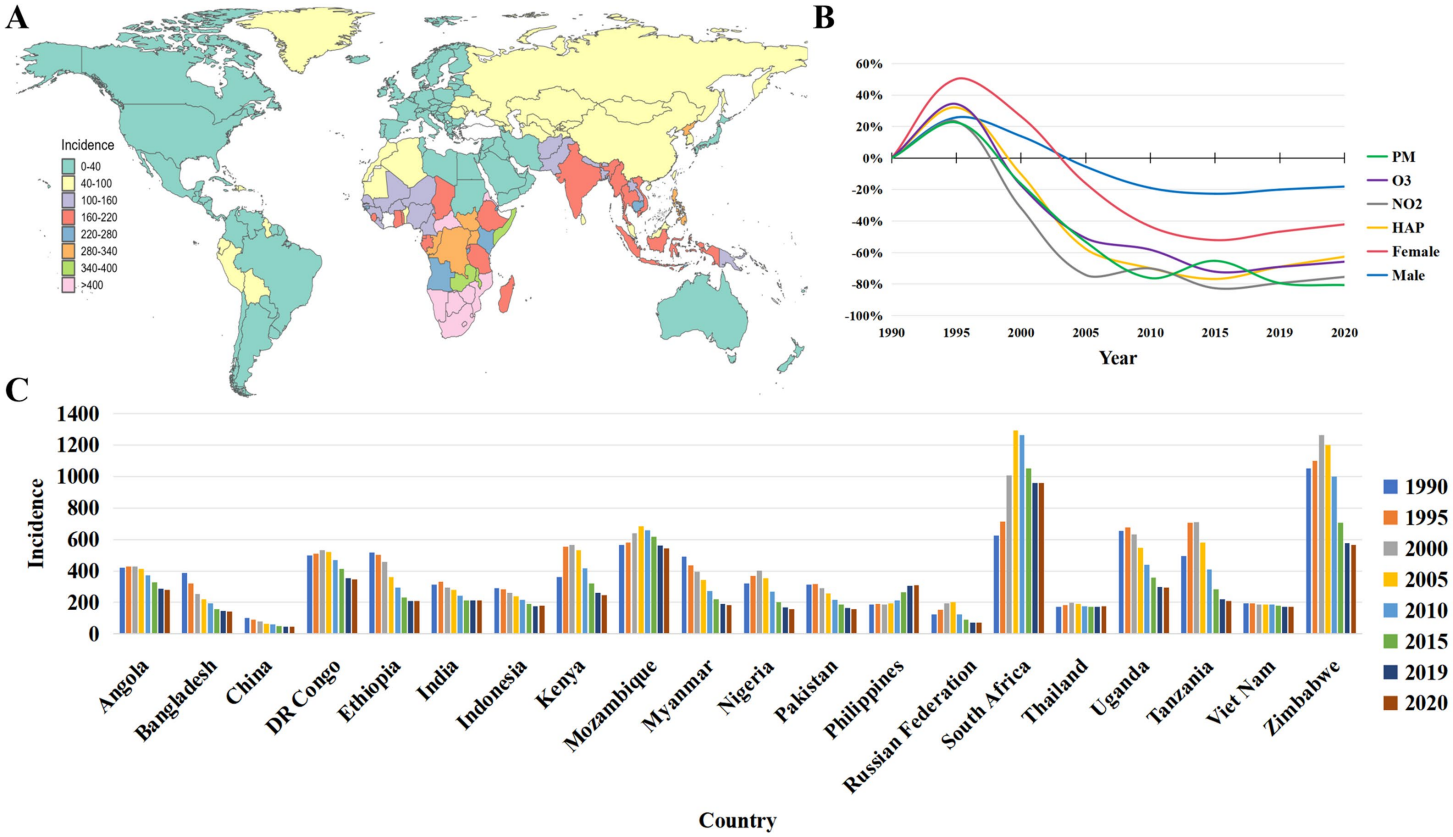
**(A)** Global incidence of tuberculosis in 2021. **(B)** Relative changes in global tuberculosis incidence (male and female) and air pollution indicators from 1990 levels, 1991–2020 (PM in the figure is ambient particulate matter pollution, O_3_ is ambient ozone pollution, NO_2_ is nitrogen dioxide pollution, HAP is household air pollution from solid fuels). **(C)** Tuberculosis temporal trends in the top 20 incidence countries.

Geographically, the majority of tuberculosis cases in 2021 were found in South-East Asia (45%), Africa (23%), and the Western Pacific (18%), with smaller proportions in the Eastern Mediterranean (8.1%), the Americas (2.9%), and Europe (2.2%). In terms of the number of new cases of tuberculosis per 100,000 people per year, the severity of the epidemic varies considerably among countries, ranging from less than 40 to more than 400 cases. In 2021, there were 47 countries with a low incidence of tuberculosis (<10 cases per 100,000 population per year), mainly in a small number of countries in the Americas, Europe, and the Eastern Mediterranean and Western Pacific regions. Most of the 30 high-burden TB countries have between 150 and 400 cases per 100,000 population, with more than 500 cases per 100,000 population in countries such as the Central African Republic, Gabon, Lesotho, the Philippines, and South Africa, as shown in Figure 1A.

The relative change in TB incidence and air pollution indicators from 1991 to 1995, based on 1990 data, showed a clear upward trend, with the rate of increase in incidence in women being the most sensitive. The relative change after 1995 generally showed a clear downward trend, with the trend of change in TB incidence in men being similar to that in women, but overall, men were more likely than women. The relative change in PM, NO_2_, and HAP all showed a downward trend in the relative amount of change, with the percentage changes in O_3_, HAP, and NO_2_ reaching a nadir around 2015 and then recovering slightly, as shown in Figure 1B.

The top 20 countries in terms of global TB prevalence in 2021 showed fluctuating prevalence rates between 1990 and 2020, with some countries showing a clear downward trend and a few countries showing an increase in prevalence. Among them, the DR Congo, Kenya, Mozambique, Nigeria, Pakistan, the Russian Federation, South Africa, Tanzania, and Zimbabwe showed a clear upward trend between 1995 and 2005, while the Philippines showed an upward trend after 2015. China, Thailand, and Vietnam showed a high prevalence in 1990 and a decreasing trend thereafter, but the rate of decline slowed down after 2000, as shown in Figure 1C.

### 3.2 Descriptive statistical analysis of air pollution and incidence of tuberculosis

The average annual incidence of TB was significantly higher in Zimbabwe and South Africa than in other countries, and the highest median values of HAP, NO_2_, O_3_, PM, and GDP were found in Uganda, Russia, India, Nigeria, and South Africa, with the highest values of 0.98, 10.44 ppb, 55.84 ppb, 61.94 $\mathrm{\mu g}/m^{3}$, and 5817.43, respectively. The distribution of TB incidence rates and factors affecting them in each country for the period 1990–2020 is shown in Table 1.

**Table 1:** Description of the average annual incidence of tuberculosis in the top 20 countries and factors influencing it.

Country	Incidence				HAP				NO_2_ (ppb)				O_3_ (ppb)				PM ($\mathrm{\mu g}/m^{3}$)				GDP			
	P_25_	M	P_75_	$\bar{x}$±s	P_25_	M	P_75_	$\bar{x}$±s	P_25_	M	P_75_	$\bar{x}$±s	P_25_	M	P_75_	$\bar{x}$±s	P_25_	M	P_75_	$\bar{x}$±s	P_25_	M	P_75_	$\bar{x}$±s
Angola	317.14	392.81	421.15	368.77 ± 63.34	0.45	0.63	0.80	0.62 ± 0.21	1.09	2.56	4.01	2.62 ± 1.59	42.78	44.70	46.64	45.31 ± 3.00	23.73	24.08	24.21	24.01 ± 0.66	851.19	1752.36	2381.89	1768.61 ± 1131.58
Bangladesh	154.72	208.46	270.61	228.00 ± 88.26	0.80	0.89	0.94	0.87 ± 0.09	3.74	4.87	5.67	4.72 ± 1.01	52.57	55.54	61.72	56.63 ± 6.52	50.83	52.85	53.49	52.39 ± 5.96	390.35	634.83	1457.52	986.39 ± 796.65
China	48.40	63.34	81.23	67.32 ± 20.74	0.39	0.59	0.76	0.58 ± 0.22	7.45	7.80	8.37	8.15 ± 1.09	48.55	49.41	51.08	50.26 ± 2.97	48.42	49.18	49.83	49.19 ± 1.56	871.94	3151.94	8548.28	4594.97 ± 4338.62
DR Congo	397.57	484.98	513.62	455.86 ± 75.25	0.94	0.97	0.98	0.96 ± 0.03	2.07	2.15	2.34	2.26 ± 0.26	39.50	41.34	44.70	41.78 ± 4.86	25.41	26.20	26.54	25.91 ± 0.83	247.75	358.73	492.72	362.73 ± 158.41
Ethiopia	226.18	328.69	469.13	347.96 ± 131.11	0.97	0.97	0.98	0.97 ± 0.01	1.99	2.13	2.18	2.14 ± 0.21	35.13	37.33	41.73	38.63 ± 4.37	25.70	28.45	30.26	28.03 ± 2.81	153.39	294.87	682.85	424.44 ± 325.59
India	213.80	259.38	299.65	261.91 ± 48.31	0.65	0.75	0.81	0.73 ± 0.10	5.34	6.05	6.75	6.01 ± 0.94	53.12	55.84	62.87	57.55 ± 6.83	58.65	61.48	62.19	61.17 ± 7.18	424.93	1030.57	1670.24	1099.17 ± 707.52
Indonesia	186.83	226.98	266.66	229.10 ± 46.07	0.31	0.45	0.52	0.43 ± 0.16	3.50	3.79	3.92	3.72 ± 0.35	30.21	34.37	37.72	33.88 ± 4.15	21.08	23.91	27.95	24.18 ± 4.56	957.83	2171.92	3465.50	2260.70 ± 1495.82
Kenya	306.33	388.55	538.15	407.30 ± 129.99	0.87	0.88	0.90	0.88 ± 0.02	2.20	2.34	2.38	2.27 ± 0.17	27.05	27.86	37.01	31.30 ± 6.34	17.85	20.10	22.36	20.27 ± 3.30	401.39	808.21	1606.60	1017.26 ± 705.25
Mozambique	565.11	599.82	643.16	606.22 ± 50.06	0.96	0.97	0.97	0.97 ± 0.01	1.36	1.80	1.99	1.71 ± 0.31	27.07	30.96	34.50	31.20 ± 4.43	19.59	20.05	20.77	20.20 ± 0.76	289.99	436.30	487.99	395.68 ± 146.10
Myanmar	212.02	307.63	404.60	316.12 ± 117.00	0.83	0.91	0.94	0.89 ± 0.07	2.86	3.32	3.54	3.21 ± 0.39	37.62	39.60	42.40	39.58 ± 2.81	35.11	39.11	40.84	37.38 ± 4.70	141.25	493.55	1242.22	662.41 ± 600.31
Nigeria	193.88	294.00	356.14	279.56 ± 94.83	0.79	0.83	0.88	0.83 ± 0.07	3.31	3.41	3.49	3.40 ± 0.15	42.66	44.78	50.47	46.44 ± 4.88	53.11	61.94	64.04	59.20 ± 6.46	566.96	1662.51	2223.16	1503.62 ± 912.25
Pakistan	180.83	236.93	296.87	237.86 ± 65.61	0.57	0.69	0.77	0.67 ± 0.12	5.72	6.04	6.59	6.31 ± 1.11	53.99	55.06	60.32	56.94 ± 4.73	54.50	58.57	59.63	57.46 ± 6.32	512.36	799.80	1292.41	871.87 ± 429.51
The Philippines	189.58	204.12	275.53	231.53 ± 53.43	0.52	0.58	0.60	0.56 ± 0.07	4.74	5.39	5.74	5.22 ± 0.78	24.60	25.64	27.29	26.04 ± 1.89	23.73	28.57	32.33	27.63 ± 5.55	1185.03	1723.53	3036.83	2021.96 ± 1062.93
Russian	85.36	124.27	162.60	128.84 ± 50.21	0.02	0.04	0.06	0.04 ± 0.02	9.89	10.44	11.48	10.99 ± 1.67	37.86	43.04	43.95	41.14 ± 3.74	12.62	15.59	21.44	17.16 ± 5.58	3285.98	7318.24	10295.56	6868.36 ± 3974.40
South Africa	896.65	982.20	1103.10	983.12 ± 233.77	0.14	0.24	0.35	0.26 ± 0.13	4.39	5.29	5.70	5.12 ± 0.67	32.94	36.09	38.95	36.22 ± 3.20	24.09	24.53	24.72	24.33 ± 0.62	3738.65	5817.43	6325.33	5361.88 ± 1758.24
Thailand	173.83	176.75	184.92	181.11 ± 9.83	0.23	0.34	0.47	0.37 ± 0.17	3.97	4.60	5.20	4.47 ± 0.81	32.48	38.39	44.53	38.41 ± 6.52	30.85	36.90	40.78	36.25 ± 6.17	2637.49	3936.31	6029.33	4325.05 ± 2323.43
Uganda	343.14	493.12	637.01	487.41 ± 160.24	0.98	0.98	0.98	0.98 ± 0.00	1.47	1.50	1.64	1.58 ± 0.15	33.14	36.46	42.08	37.44 ± 5.17	30.74	31.56	32.66	31.75 ± 2.43	273.19	576.87	830.25	558.79 ± 301.60
Tanzania	267.10	451.94	610.08	451.12 ± 203.57	0.97	0.97	0.98	0.98 ± 0.01	1.77	1.88	1.94	1.85 ± 0.13	27.01	29.51	35.85	31.21 ± 5.39	17.20	21.53	23.18	20.81 ± 3.67	343.82	605.54	957.13	622.19 ± 366.83
Vietnam	176.76	185.52	188.13	183.11 ± 8.61	0.39	0.59	0.81	0.61 ± 0.24	4.60	5.94	7.33	5.95 ± 1.58	33.91	38.48	38.84	36.88 ± 3.23	23.40	26.42	27.69	25.31 ± 3.34	366.22	1188.60	2819.20	1602.79 ± 1453.76
Zimbabwe	672.99	1025.60	1123.17	932.36 ± 277.25	0.69	0.70	0.72	0.71 ± 0.04	2.50	2.57	2.62	2.55 ± 0.12	34.27	38.31	39.82	37.52 ± 3.72	20.37	20.80	21.46	21.17 ± 1.28	626.44	903.17	1382.10	961.77 ± 394.33

### 3.3 Analysis of the correlation between air pollution and annual incidence of tuberculosis

The correlation coefficients of the influencing factors and the TB incidence rates of the top 20 countries in the global TB incidence rate in 2021 for the period 1990–2020 were obtained by Spearman correlation analysis and are shown in Table 2. The results show that the correlation between the TB incidence rate and the influencing factors is statistically significant (*p* < 0.05). HAP is positively correlated with the TB incidence rate (r_HAP_ = 0.476), and NO_2_, O_3_, PM, and GDP were negatively correlated with TB incidence (r_NO2_ = −0.622, r_O3_ = −0.419, r_PM_ = −0.323, r_GDP_ = −0.477), which suggests that TB incidence may decrease as HAP decreases and as NO_2_, O_3_, PM, and GDP increase. NO_2_ had the most significant effect on TB incidence.

**Table 2:** Results of correlation analyses between tuberculosis incidence and factors influencing it in various countries, 1990–2020.

	Incidence	HAP	NO_2_	O_3_	PM
HAP	0.476^**^				
NO_2_	−0.622^**^	−0.745^**^			
O_3_	−0.419^**^	−0.169^*^	0.394^**^		
PM	−0.323^**^	0.040	0.316^**^	0.596^**^	
GDP	−0.477^**^	−0.844^**^	0.536^**^	0.204^**^	−0.060

*p < 0.05, **p < 0.01.

### 3.4 Impact of single-influence factors on the incidence of TB

According to the results in Table 2, four air pollutant factors (HAP, NO_2_, O_3_, PM, and GDP) and one factor (economic development level) were used as explanatory variables, and the incidence of tuberculosis was used as a response variable. A model was constructed using a spline smoothing function with the five explanatory variables to analyse the significance of the influence of each explanatory variable on the response variable and the goodness-of-fit of the model, as shown in Table 3. All factors had significant effects on the change in TB incidence at a *p* value of <0.001, indicating that each factor was statistically significant when used alone as an explanatory variable for the change in TB incidence. PM had a high model explanatory rate of the change in TB incidence (27.0%), O_3_ had a low model explanatory rate of the change in TB incidence (18.1%), and the model explanatory rates of the other influencing factors were all above 20% (21.5–24.8%), and the R^2^ of each factor was small (0.163–0.240), indicating that the model fit and explanatory power of the effect of each explanatory variable alone on the change in TB incidence were poor. Although the model fit and explanatory power of each influencing factor as a separate explanatory variable to construct the effect on the change in TB incidence rate were poor, they were all statistically significant (*p* < 0.001).

**Table 3:** Tuberculosis incidence and the results of fitting a GAM model with a single influencing factor.

FACTOR	Smooth terms	RDF	*F* value	*P* value	R^2^	DVE	GCV	AIC
HAP	7.992	8.721	4.555	<0.001	0.175	21.7	59416.700	2214.308
NO_2_	6.522	7.640	6.110	<0.001	0.215	24.8	55977.36	2204.928
O_3_	3.453	4.276	7.538	<0.001	0.163	18.1	58547.210	2212.348
PM	6.383	7.537	6.893	<0.001	0.240	27.0	54190.210	2199.750
GDP	5.669	6.830	5.487	<0.001	0.186	21.5	57764.880	2210.036

### 3.5 Multi-influential factors on the incidence of tuberculosis

The elements with statistical significance in the one-way analysis were used as explanatory variables, and the TB incidence rate was used as a response variable. GAMs were fitted between multiple factors and the TB incidence rate, and the model used the logarithmic function as a linking function. The smoothing term was used as a spline function, and the results of the fitting are shown in Table 4. The results of the multifactor fitting showed that there was no statistical significance of PM (*p* > 0.05), O_3_, and NO_2_ were at a *p* value of <0.05, HAP and GDP significantly affected the changes in TB incidence at a *p* value of <0.001, and the R^2^ of the fitted equation was 0.918, with a variance explained rate of 94.2%, which indicated that the model was well fitted. The four statistically significant influencing factors had a high rate of explaining the changes in TB incidence, which indicated that HAP, GDP, NO_2_, and O_3_ had a significant influence.

**Table 4:** Tuberculosis incidence and multi-influence factor GAMs fitting results.

Smoothing effects term	Original model					Adjusted model				
	VIF	EDF	RDF	*F* value	*P* value	VIF	EDF	RDF	*F* value	*P* value
HAP	1.754	1.000	1.000	15.482	<0.001	1.714	1.000	1.000	13.729	<0.001
NO_2_	2.978	4.932	5.567	2.602	0.019	2.939	5.131	5.696	3.067	0.007
O_3_	3.485	9.537	10.901	2.217	0.022	3.994	4.564	5.307	2.506	0.030
PM	4.229	1.583	1.822	2.666	0.146	.	.	.	.	.
GDP	2.485	10.206	11.350	3.763	<0.001	2.135	4.091	4.900	4.465	0.001

PM was statistically significant in independent analysis; however, it was not statistically significant in the multifactor analysis (*p* < 0.05). It was initially hypothesized that there might be covariance among the influencing factors, so the diagnosis of covariance was carried out, and the result showed that the value of the variance inflation factor (VIF) of PM was 4.229. In general, no significant covariance can be considered when VIF < 5. Although PM showed relatively strong covariance compared to the other four air pollutant factors, it still belonged to the weak covariance in light of the judgement criteria, so covariance was not the cause of no statistical significance in the multifactorial analysis. Although PM showed relatively strong covariance compared with the other four air pollutants, it still had weak covariance in terms of the judgement criterion; therefore, covariance was not the main reason for the lack of statistical significance in the multifactor analysis. On further analysis, it is more likely that the small range of PM itself has weakened its effect on the results, which leads to inconsistency between the results of the independent analysis and the multifactor analysis. After further deleting the influencing factor PM, the results of reconstructing the GAMs model with multiple influencing factors showed that all explanatory variables in the fitted equations were statistically significant (*p* < 0.01), and there was a significant non-linear relationship between them and changes in TB incidence (EDF and RDF were both ≥1), with a model R^2^ of 0.905 and a variance explained rate of 92.7%, suggesting that the multifactorial GAMs fit was overall higher than the fitting effect of a single factor on the change in TB incidence, which indirectly reflected that the change in TB incidence was affected by multiple influencing factors. The degree of influence on the change in TB incidence rate in the multifactorial GAMs was HAP > GDP > NO_2_ > O_3_ in descending order, suggesting that HAP is the key factor influencing the change in TB incidence rate.

### 3.6 Diagnosis of effects of changes in morbidity

**Figure 2:**
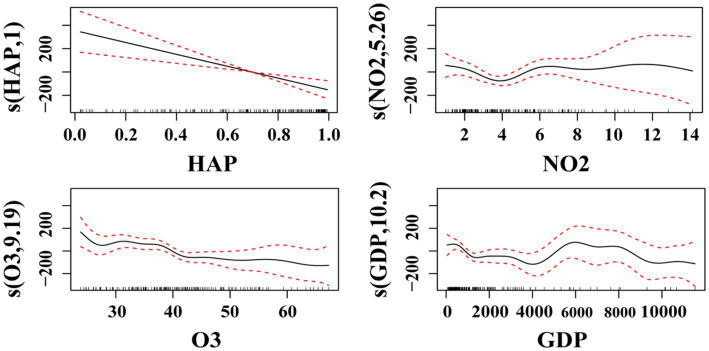
Effect of factors on the incidence of tuberculosis (HAP in the figure is household air pollution from solid fuels, NO_2_ is nitrogen dioxide pollution, O_3_ is ambient ozone pollution, and GDP is gross domestic product).

The GAMs model was built between multiple factors and TB incidence to obtain the smooth regression function of the explanatory variables and to obtain the effect plot of the influence of each influencing factor on TB incidence (Figure 2). The horizontal axis represents each air pollutant factor, and the unit of each air pollutant factor is the same as that of the data source section; the vertical axis represents the value of the smoothing function of each air pollutant factor, and the number in parentheses denotes the estimated degree of freedom (df); the black solid line in the figure is the smoothed fitting curve of the variable and the incidence rate, which can reflect the correlation tendency of the two, and the red dashed line is the 95% confidence interval of the fitting curve, which can reflect the uncertainty of the effect. The results in Figure 2 show that there was a linear relationship between HAP and TB incidence, and although the actual data might fluctuate or deviate slightly from the straight line, in general, they were fitted to a straight line. The linear relationship implies that the incidence of TB decreases with increasing HAP, whereas the relationships between NO_2_, O_3_, GDP, and TB incidence are non-linear. The incidence of TB increases monotonically with NO_2_ above 4 ppb and also tends to increase slightly when NO_2_ is around 8.5 ppb. When the O_3_ concentration is higher than approximately 25, the incidence rate of tuberculosis increases significantly with the increase in O_3_ concentration; after that, the effect of O_3_ concentration on the incidence rate of tuberculosis tends to level off with the increase in O_3_ concentration; when the O_3_ concentration is higher than approximately 37, the incidence rate of tuberculosis decreases significantly with the increase in O_3_ concentration; and the change in the incidence rate of tuberculosis is basically unaffected by the increase in O_3_ concentration above approximately 42. GDP When the GDP is lower than about 1,500, the incidence rate of tuberculosis decreases significantly with the increase in GDP; after that, the effect of GDP on the incidence rate of tuberculosis tends to flatten out with the increase in GDP; when the GDP is higher than about 3,200, the incidence rate of tuberculosis shows a decreasing tendency with the increase in GDP; when the GDP is higher than about 4,000–6,000, the incidence rate of tuberculosis shows a significant increasing tendency. When the GDP is greater than 6,000, the impact on the change of tuberculosis incidence rate tends to level off again; when it is greater than 8,000, the tuberculosis incidence rate decreases significantly as the GDP continues to rise; when the GDP is as high as about 9,200, it has basically no impact on the change of the tuberculosis incidence rate.

Table 5 shows that for different countries, the importance of the factors varied significantly. Among them, Pakistan and South Africa suggest that NO_2_ is the key factor influencing the change in TB incidence; HAP is the key factor influencing the change in TB incidence in Angola and Thailand; for China, the Philippines, and Tanzania, the key factor is GDP; in Ethiopia, India, Kenya, and Russia, PM is the key factor influencing the change in TB incidence; and the key factor influencing the change in TB incidence in nine countries, including Bangladesh, is O_3_. From the results of the optimal model fitting, the R^2^ and DVE of Angola, Indonesia, Mozambique, the Philippines, and Zimbabwe had the highest R^2^ and DVE of 1.000 and 100%, respectively, followed by Nigeria with 0.999 and 100%, respectively, while the optimal models for the rest of the countries had R^2^ values ranging from 0.835 to 0.998 and DVE values ranging from 97.7 to 99.9%. The optimal model AIC for all countries ranges from −108.35 to 110.97. Notably, South Africa had the lowest R^2^ and DVE of −0.542 and 83.8%, respectively, and negative R^2^ values strongly suggest the presence of strong, unobserved, or unmodelled confounding factors (e.g., specific epidemiological factors, socio-structural factors, or data quality issues) that dominate the temporal trends in TB incidence in South Africa. As shown in Figure 1C, South Africa’s TB incidence data suffered from more significant and irregular annual fluctuations than those of the other countries studied. The GAMs, while providing a better fit to the nonlinear relationship, were still sensitive to extreme values. Significant and irregular annual fluctuations may have caused the incidence rates to deviate from the trend explained by the model based on the selected air pollutant factors versus GDP. These uncaptured fluctuations increase the residual sum of squares. If this residual sum greatly exceeds the total sum of squares, R^2^ becomes negative. Thus, South Africa’s negative R^2^ stems from strong unmeasured confounders and high data volatility, indicating unique epidemic drivers requiring further study.

**Table 5:** Results of optimal model fit and ranking of importance of influencing factors by country from 1990 to 2020.

Country	Influencing factors in descending order of importance	R^2^	DVE (%)	GCV	AIC
Angola	HAP, NO_2_, O_3_, GDP, PM	1.000	100	25.05	−108.35
Bangladesh	O_3_, NO_2_, GDP, PM, HAP	0.988	99.9	948.76	57.04
China	GDP, O_3_, HAP, NO_2_, PM	0.983	99.8	75.73	36.01
DR Congo	O_3_, GDP, NO_2_, PM, HAP	0.954	99.6	3100.20	64.15
Ethiopia	PM, NO_2_, GDP, HAP, O_3_	0.997	100	409.02	51.73
India	PM, GDP, NO_2_, O_3_, HAP	0.835	97.7	3142.00	69.61
Indonesia	O_3_, GDP, PM, NO_2_, HAP	1.000	100	375.59	−91.96
Kenya	PM, O_3_, NO_2_, GDP, HAP	0.949	99.3	7252.50	75.83
Mozambique	O_3_, HAP, NO_2_, PM, GDP	1.000	100	866.67	−20.70
Myanmar	O_3_, NO_2_, GDP, PM, HAP	0.998	100	178.66	46.38
Nigeria	O_3_, GDP, HAP, PM, NO_2_	0.999	100	75.12	39.66
Pakistan	NO_2_, HAP, O_3_, PM, GDP	0.992	99.9	264.55	49.84
Philippines	GDP, HAP, PM, NO_2_, O_3_	1.000	100	166.82	−96.72
Russian	PM, HAP, O_3_, GDP, NO_2_	0.995	99.9	118.08	41.61
South Africa	NO_2_, PM, GDP, HAP, O_3_	−0.542	83.8	916690.00	110.97
Thailand	HAP, NO_2_, GDP, PM, O_3_	0.997	100	7.15	6.75
Uganda	O_3_, GDP, PM, NO_2_, HAP	0.997	100	685.16	56.67
Tanzania	GDP, NO_2_, O_3_, PM, HAP	0.994	99.9	2423.50	65.59
Vietnam	O_3_, PM, HAP, GDP, NO_2_	0.971	99.6	17.49	28.32
Zimbabwe	O_3_, PM, GDP, NO_2_, HAP	1.000	100	3228.30	−100.08

### 3.7 Interaction of influencing factors on the development of tuberculosis

Changes in tuberculosis incidence rate are affected by a variety of influencing factors, and the interaction between the explanatory variables has an obvious influence on changes in tuberculosis incidence rate. The GAMs model was constructed two by two between the explanatory variables to analyse the effect of the interaction terms on the changes in TB incidence rates, which is conducive to an in-depth understanding of the effects of the factors on the changes in TB incidence rates, and the results are shown in Table 6.

**Table 6:** Results of GAM model fit for interaction of influencing factors with TB incidence rate.

Interaction term	EDF	RDF	*F* value	*P* value
HAP-NO_2_	10.820	14.09	2.430	0.005
HAP-O_3_	1.000	1.00	3.558	0.062
HAP-GDP	8.719	11.39	2.179	0.021
NO_2_-O_3_	4.921	27.00	0.337	0.046
NO_2_-GDP	<0.001	27.00	0.000	0.007
O_3_-GDP	<0.001	27.00	0.000	0.007

It can be seen that the degree of freedom of the interaction term HAP-O_3_ is 1, indicating that there is a linear relationship between interaction and TB incidence, and the degree of freedom of each interaction term in the interaction terms HAP-NO_2_, HAP-GDP, and NO_2_-O_3_ is greater than 1, indicating that there is a non-linear relationship between the interaction term and TB incidence; while the effective degree of freedom of NO_2_-GDP and O_3_-GDP is <0.001, which from the conceptual logic tends to 0, indicating that there is almost no meaningful linear or non-linear relationship between the interaction and TB incidence, and the contribution in the model is weak. The R^2^ of the GAM model for the interaction of influencing factors and TB incidence was 0.917, and the DVE was 94.1%, indicating that the model fit was good and the interaction term explained a high percentage of the change in TB incidence; the five cross terms in the fitted equations, namely, HAP-NO_2_, HAP-GDP, NO_2_-O_3_, NO_2_-GDP, and O_3_-GDP, were statistically significant, indicating that their contribution to TB incidence was significant at a *p* value of <0.05 and significantly affects the change in TB incidence rate.

**Figure 3:**
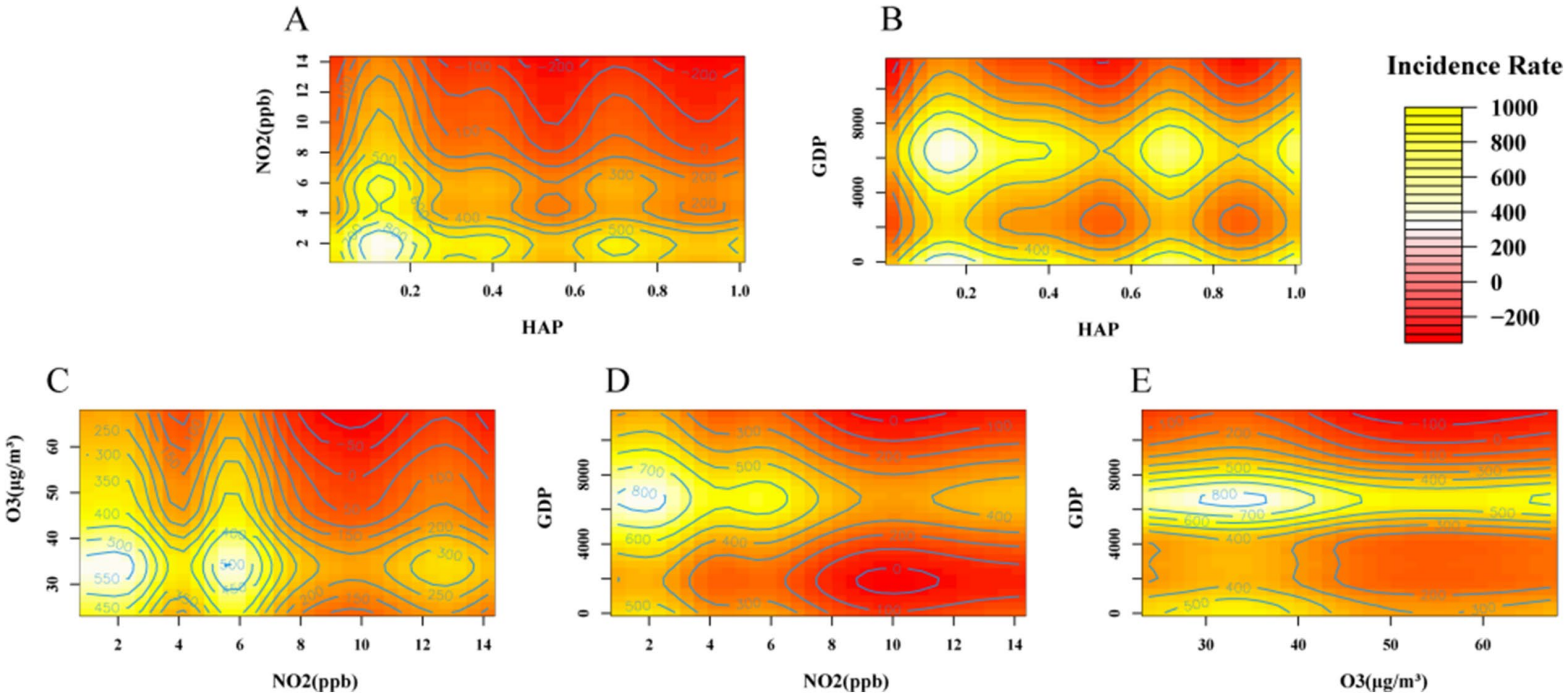
Interaction effects of influencing factors on the impact of changes in tuberculosis incidence rates, the risk of TB increased at 2–6 ppb for NO_2_ from **(A,C,D)**, **(B, E)** shows that when GDP is approximately 4,000–6,000, the incidence risk of TB also tends to increase. and the synergistic effects of the factors on TB incidence are detailed above. (HAP in the figure is household air pollution from solid fuels, NO_2_ is nitrogen dioxide pollution, GDP is gross domestic product, and O_3_ is ambient ozone pollution).

The statistically significant impact factor interaction model was visualized and plotted in Figure 3 in order to analyse the characteristics of the simultaneous changes in the response variable TB incidence across the dimensions of the explanatory variables. As can be seen from the figure, there are many ‘wavy’ contours, which means that it has a higher fit to the dependent variable and a lower smoothing factor. In the effect of NO_2_ interacting with HAP, O_3_, and GDP on TB incidence, the risk of TB increased at 2–6 ppb and above 12 ppb, but the change in TB incidence varied in the interaction of NO_2_ with other factors, and in general, there was a synergistic effect between the increase in TB incidence by HAP, O_3_, and GDP and NO_2_. Overall, HAP, O_3_, GDP, and NO_2_ had a synergistic effect on the increase in TB incidence, i.e., TB incidence tended to increase with increasing NO_2_ at HAP < 0.2; the risk of TB incidence increased at lower NO_2_, especially at 2–6 ppb, and O_3_ at <40; and TB incidence also tended to increase with increasing NO_2_ at a GDP of about 4,000–6,000.

## 4 Discussion

Tuberculosis (TB) re-emerged as the leading cause of death from a single infectious agent in the world in 2023, and the number of TB cases globally has been increasing every year since 2020, with more and more countries and regions focusing on TB surveillance and early warning due to the enormous harm and social impact of TB. This study analysed information on the incidence of TB in 2021 in various countries globally and found that the severity of TB epidemics in 2021 varied greatly among countries globally, and the reasons for the differences may include the varying severity of the impact of the COVID-19 pandemic on individual countries (37–39). Indoor air quality has a significant effect on the respiratory tract (40), and air pollution was the second most important risk factor leading to deaths in the world in 2021 (6). Worldwide, more than 4 million people die each year from indoor air pollution. This largely preventable exposure is a key target for reducing morbidity and mortality worldwide (41). Therefore, this study, by analysing the relative amount of change in TB incidence and air pollution indicators from 1991 to 2020 based on 1990 data, found that the relative amount of change in TB incidence and air pollution indicators based on 1990 since 1995 has generally shown a clear downward trend. Since the air pollutant factor NO_2_ disrupts respiratory clearance, suppresses antimicrobial immunity, and promotes chronic inflammation; O_3_ oxidatively damages lung tissues, weakens immune cell recruitment, and exacerbates neutrophil-mediated tissue destruction; PAHs (e.g., BaP) produced by solid fuel pollution disrupt immune homeostasis, and concomitant production of CO results in hypoxic immune suppression; and PM is primarily responsible for the development of TB infection through inhibition of the macrophage bactericidal function, interfering with T-cell immunity and inducing chronic inflammation, significantly increasing the risk of TB infection. Therefore, the decreasing trend may be due to the improvement in air pollution indicators, which is closely related to the implementation of environmental protection policies (42). Meanwhile, the decline in TB incidence may be related to sustained public health interventions and socio-economic development by the World Health Organization and other countries. The incidence rate in men has been higher than that in women, which may be related to biological differences, social behavioral differences, or occupational exposure differences. The results of Spearman’s correlation analysis showed that the incidence rate of tuberculosis had a significant correlation with the influencing factors of HAP, NO_2_, O_3_, PM, and GDP (*p* value of <0.01). This is consistent with the findings of Alkhanani MF, who found that nitrogen dioxide levels were significantly associated with an increased incidence of tuberculosis (24). The analysis shows a positive correlation between TB incidence and HAP (rHAP >0), which is consistent with the findings of Lee et al. (19). At the same time, the incidence of tuberculosis was negatively correlated with all other variables (rs < 0), which is consistent with the negative correlation between O_3_ and TB outpatient cases derived by Huang et al. (43); and also with the negative correlation between NO_2_ and the risk of drug-resistant TB incidence found by Yao et al. (44). This is also consistent with the finding of Feng et al. (21) that the risk of PTB is negatively correlated with O_3_ exposure. However, this is inconsistent with the findings of Zhao et al. (45) who found that for every 10 $\mathrm{\mu g}/m^{3}$ increase in NO_2_ concentration, there was an 11.4% increase in the risk of death in newly treated TB patients (45), and a study in Chengdu found a positive correlation between outdoor PM_10_ and NO_2_ exposures and the number of TB cases (34), in contrast to the findings (26).

Since there is considerable variation in risk exposure between socioeconomic factors, especially in areas affected by violence and conflict (46–48), four air pollution indicators (HAP, NO_2_, O_3_, and PM) were included in the construction of a single-influence GAMs model for changes in TB incidence rates with the indicator of the level of economic development, GDP, and all five influences significantly affected TB incidence changes at a *p* value of <0.001. They significantly influenced changes in TB incidence, with PM explaining the most variance in changes in TB incidence, followed by NO_2_, HAP, GDP, and O_3_ explaining less variance. The four factors, HAP, GDP, NO_2_, and O_3_, significantly influenced the changes in TB incidence rates at a *p* value of <0.05 in the multi-influence GAMs model. Among the top 20 countries in terms of TB incidence in 2021, the differences in the importance of the influencing factors were more obvious, and the key factors of TB incidence were not exactly the same in each country. However, the variance explained rate was higher than 99% in each country except South Africa, and R^2^ and DVE were the lowest in South Africa, which indicated that other than air pollutants and GDP, other factors had a somewhat greater influence on changes in TB incidence in South Africa than in other countries. The GAMs model was used to further analyse the interaction effects of the main influencing factors on the changes in TB incidence rates, and the results showed that the five interaction terms, HAP-NO_2_, HAP-GDP, NO_2_-O_3_, NO_2_-GDP, and O_3_-GDP, all significantly influenced the changes in TB incidence rates at a *p* value of <0.05. Confounding factors such as population density and health care resources were not adjusted for in this study because the GBD data already included sociodemographic covariates. Combined with the visualization of the interaction effects, it was found that there was a synergistic amplification of the increase in TB incidence by HAP, O_3_, GDP, and NO_2_; more specifically, at HAP < 0.2, TB incidence tended to increase with increasing NO_2_, and at lower NO_2_, especially at 2–6 ppb, the risk of TB incidence increased at O_3_ at less than 40, suggesting that low NO_2_ concentrations would lead to an increase in TB incidence, and were higher at concentrations of 2–6 ppb. Low HAP as well as high O_3_ conditions favoured TB. In addition, at a GDP of about 4,000–6,000, TB incidence tended to increase as NO_2_ increased, suggesting that the air pollutant factor dominated the effect of GDP interacting with HAP, NO_2_, and O_3_ on TB incidence. The results of this study suggest that despite the decreasing trend in the relative change in air pollution from 1990 to 2020, the TB incidence in each country is still adversely affected by air pollution to varying degrees. Household air pollution from solid fuels was the key factor influencing the change in TB incidence in Angola and Thailand. The newly introduced air pollution risk factor NO_2_ in GBD 2021 had a significant effect on the change in TB incidence in Pakistan and South Africa, and PM had a significant effect on the change in TB incidence in Ethiopia, India, Kenya, and Russia. PM had a greater impact on the change in TB incidence than other air pollutants in four countries, and O_3_ had a significant impact on the change in TB incidence in nine countries, including Bangladesh.

This study has certain limitations. First, there are many factors affecting the incidence of tuberculosis, and this study only conducted a preliminary investigation from the perspective of air pollution and economic development. More factors, such as sex, age, occupation, and meteorological factors, will be considered in the follow-up study to more comprehensively analyse the key factors affecting the incidence of tuberculosis in countries with a high incidence of tuberculosis. Second, only the top 20 countries in terms of tuberculosis incidence in 2021 were selected for this study, mostly in Africa, Asia, and Europe, and in future studies, comparisons should be made with the middle and bottom 20 countries in terms of incidence so as to make the conclusions more representative.

## 5 Conclusion

Based on our study, each air pollutant has a more or less significant effect on the incidence of TB, while there is a synergistic amplification of the increase in TB incidence by HAP, O_3_, GDP, and NO_2_, with low NO_2_ concentrations leading to an increase in the incidence of TB, and higher at concentrations of 2–6 ppb. Low HAP as well as high O_3_ conditions favour TB. Air pollutants play a role in the incidence and prevalence of TB in the population, not necessarily directly but perhaps indirectly, by influencing the distribution of TB in the population by affecting various aspects of morbidity (living habits of the population, routes of transmission, and body mass of susceptible people themselves). Therefore, as countries progress economically, the implementation of sustainable development policies and investment in air pollution control strategies can reduce the long-term negative impacts of air pollution on the health of the population. If countries are able to target air pollution policies to further reduce exposure to air pollution, it may go a long way in assisting the preventive treatment of TB and thus reduce TB incidence. It is hoped that the findings of this study will provide a scientific reference for the top 20 TB incidence countries in air pollution interventions at the national level.

## Synopsis

The influence of various air pollution indicators on TB incidence varies from country to country. Therefore, this study may provide a reference for national TB prevention policies.

## Data Availability

Publicly available datasets were analyzed in this study. This data can be found at: https://ghdx.Healthdata.org/ihme_data.

## Glossary

NO_2_: The full name is nitrogen dioxide pollution: The atmospheric pollutant NO_2_ acts as a precursor to near-surface O_3_ pollution and may contribute to TB progression through multiple pathways.
O_3_: The full name is ambient ozone pollution: O_3_ exposure deteriorates lung function, exacerbates airway inflammatory responses, affects lung ventilation, and increases the risk of tuberculosis.
PM: The full name is ambient particulate matter pollution: PM particles are small, light, and toxic, and may absorb more harmful substances. These are the important risk factors for TB infection.
HAP: The full name is household air pollution from solid fuels: the core source of indoor air pollution is incomplete combustion of solid fuels, which produces black carbon particles and PAHs, which have stronger oxidative stress-inducing capacity and endothelial dysfunction effects compared to PM.
GDP: The full name is gross domestic product: GDP provides an indication of the level of economic development.
GAMs: The full name is generalized additive models: GAMs are extensions of generalized linear models (GLM) that combine the interpretability of linear models with the flexibility of nonparametric models for dealing with complex nonlinear data relationships.
EDF: The full name is effective degrees of freedom: it measures the complexity of the nonlinear terms in the model, which can reflect the flexibility of the fit.
RDF: The full name is residual degrees of freedom: a measure of the degrees of freedom remaining after model fitting, which can reflect data redundancy.
DVE: The full name is deviance explained: DVE indicates that the variance of the response variable explained by the model accounts for the proportion of the total variance.
GCV: The full name is generalized cross-validation value: GCV is a trade-off indicator between the complexity and goodness of fit.
AIC: The full name is Akaike Information Criterion: AIC weighs the complexity of the estimated model against the goodness of fit of this model to the data.
VIF: The full name is variance inflation factor: the strength of multicollinearity between explanatory variables can be detected.
